# Celebrex Adjuvant Therapy on Coronavirus Disease 2019: An Experimental Study

**DOI:** 10.3389/fphar.2020.561674

**Published:** 2020-11-06

**Authors:** Wenxin Hong, Yan Chen, Kai You, Shenglin Tan, Feima Wu, Jiawang Tao, Xudan Chen, Jiaye Zhang, Yue Xiong, Fang Yuan, Zhen Yang, Tingting Chen, Xinwen Chen, Ping Peng, Qiang Tai, Jian Wang, Fuchun Zhang, Yin-Xiong Li

**Affiliations:** ^1^ Institute of Public Health, Guangzhou Institutes of Biomedicine and Health, Chinese Academy of Sciences, Guangzhou, China; ^2^ The Eighth People's Hospital of Guangzhou, Guangzhou, China; ^3^ Guangdong Provincial Key Laboratory of Biocomputing, Guangzhou Institutes of Biomedicine and Health, Chinese Academy of Sciences, Guangzhou, China; ^4^ University of Chinese Academy of Sciences, Beijing, China; ^5^ Guangzhou Institute of Cardiovascular Disease, Guangdong Key Laboratory of Vascular Diseases, State Key Laboratory of Respiratory Disease, The Second Affiliated Hospital, Guangzhou Medical University, Guangzhou, China; ^6^ Guangzhou Regenerative Medicine and Health Guangdong Laboratory, Guangzhou, China; ^7^ The First Affiliated Hospital, Sun Yat-sen University, Guangzhou, China

**Keywords:** COVID-19, COX-2, prostaglandins, PGE_2_, celebrex

## Abstract

**Background:** The pandemic of coronavirus disease 2019 (COVID-19) resulted in grave morbidity and mortality worldwide. There is currently no effective drug to cure COVID-19. Based on analyses of available data, we deduced that excessive prostaglandin E_2_ (PGE_2_) produced by cyclooxygenase-2 was a key pathological event of COVID-19.

**Methods:** A prospective clinical study was conducted in one hospital for COVID-19 treatment with Celebrex to suppress the excessive PGE_2_ production. A total of 44 COVID-19 cases were enrolled, 37 cases in the experimental group received Celebrex as adjuvant (full dose: 0.2 g, *bid*; half dose: 0.2 g, *qd*) for 7–14 days, and the dosage and duration was adjusted for individuals, while seven cases in the control group received the standard therapy. The clinical outcomes were evaluated by measuring the urine PGE_2_ levels, lab tests, CT scans, vital signs, and other clinical data. The urine PGE_2_ levels were measured by mass spectrometry. The study was registered and can be accessed at http://www.chictr.org.cn/showproj.aspx?proj=50474.

**Results:** The concentrations of PGE_2_ in urine samples of COVID-19 patients were significantly higher than those of PGE_2_ in urine samples of healthy individuals (mean value: 170 ng/ml vs 18.8 ng/ml, *p* < 0.01) and positively correlated with the progression of COVID-19. Among those 37 experimental cases, there were 10 cases with age over 60 years (27%, 10/37) and 13 cases (35%, 13/37) with preexisting conditions including cancer, atherosclerosis, and diabetes. Twenty-five cases had full dose, 11 cases with half dose of Celebrex, and one case with ibuprofen. The remission rates in midterm were 100%, 82%, and 57% of the full dose, half dose, and control group, respectively, and the discharged rate was 100% at the endpoint with Celebrex treatment. Celebrex significantly reduced the PGE_2_ levels and promoted recovery of ordinary and severe COVID-19. Furthermore, more complications, severity, and death rate were widely observed and reported in the COVID-19 group of elders and with comorbidities; however, this phenomenon did not appear in this particular Celebrex adjunctive treatment study.

**Conclusion:** This clinical study indicates that Celebrex adjuvant treatment promotes the recovery of all types of COVID-19 and further reduces the mortality rate of elderly and those with comorbidities.

## Introduction

Severe acute respiratory distress syndrome (ARDS) caused by SARS-CoV, MERS-CoV, and SARS-CoV-2 infections is a major factor of mortality. It has been found that the nucleocapsid protein (N) and spike glycoprotein (S) of SARS-CoV can directly bind to the promoter of cyclooxygenase-2 (COX-2) gene, which drives overexpression of COX-2 in a dose-dependent manner (Yan et al., 2006; Liu et al., 2007). Homologous analysis showed that there were 90.6 and 75.8% similarity of the N and S proteins between SARS-CoV and SARS-CoV-2. Therefore, it is possible that the SARS-CoV-2 infection might also hold the potential to induce COX-2 overexpression in lung epithelial cells, resulting in a significant accumulation of prostaglandins, especially prostaglandin E_2_ (PGE_2_).

Excessive PGE_2_ levels may participate in coronavirus disease 2019 (COVID-19) pathology with one of the following mechanisms: 1) binding to the EP2 receptor, causing fever, pain, acute inflammation, and enhanced vascular permeability; 2) binding to the EP3 receptor, leading to edema, inflammatory mucus secretion, increased viscosity of exudates covered alveoli, and bronchioles blockage, thus hindering blood oxygen exchange (Morimoto et al., 2014); 3) binding to the EP4 receptor, causing bronchial contractions and spasms (Safholm et al., 2013), increasing airway resistance, causing respiratory and hemodynamic disorders, ARDS, and multi-organ failures; 4) inhibition of T-lymphocyte functionality, by promoting amplification, differentiation, and proliferation of Th1 and Th17 subtypes through EP4 receptor causing contact hypersensitivity of bronchioles (Niwa et al., 2009); 5) PGE_2_ and thromboxane A2 activate platelet aggregation and thrombosis, contributing to pulmonary hypertension in ischemia-reperfusion lung injury (Zamora et al., 1985).

Under the circumstances of this international emergency and the situation of having no effective drugs for COVID-19, it makes great sense to explore the pathological mechanisms of COVID-19 and to establish an integrated strategy for diagnosis and treatment with available drugs using the discovered key pathological target(s).

Here, we propose that excessive PGE_2_ may be a key in the pathology of COVID-19 and that COX-2 is the critical target for therapy. To test this hypothesis, the urinary PGE_2_ levels were determined in COVID-19 patients to verify its correlation with disease status. And, Celebrex, a specific inhibitor of COX-2, was to be used for experimental study.

## Methods

### Study Design and Participants

This was a prospective study done at Guangzhou Eighth People’s Hospital. Patients with SARS-CoV-2 infection were confirmed by next-generation sequencing or real-time Reverse transcription-polymerase chain reaction (RT-PCR) according to a previously published protocol (Huang et al., 2020). Based on the “Diagnosis and Treatment Guideline for COVID-19” of China, the clinical criteria for classification of OVID-19 stage in Chinese Guideline are listed as following. Mild: the clinical symptoms are mild, and no pneumonia manifestation can be found in CT imaging; ordinary: fever and respiratory tract symptoms, etc., and pneumonia manifestation can be seen in CT imaging; severe: meeting any of the following: 1) respiratory distress, RR ≥ 30 breaths/min; 2) oxygen saturation ≤93% at a rest state; and 3) arterial partial pressure of oxygen (PaO2)/oxygen concentration (FiO2) ≤300 mmHg; and critical: meeting any of the following: 1) respiratory failure occurs and mechanical ventilation is required; 2) shock occurs; and 3) complicated with other organ failure that requires intensive care unit care.

A total of 44 confirmed COVID-19 patients, who were admitted to hospital from January 23 to February 15, 2020, were enrolled into this study (Table 1; Supplementary Table S1). One of the 44 patients were classified as critical case (2.2%), seven of the 44 patients were classified as severe type cases (16%), and the others were ordinary type cases (81.8%). The enrolled patients were fully aware of the purpose, benefits, and potential risks, and signed the informed consent prior to this study. This investigational study design was approved by the Medical Ethics Committee of Guangzhou Eighth People's Hospital (Approve number: AF/sc-02/01.6). The study was registered on the Chinese Clinical Trials Registry, ChiCTR2000031630.

**TABLE 1:** Characteristics and clinical outcomes of COVID-19 patients.^a^

Parameters	All patient (n = 43),^b^ *n* (%)	Celebrex (oral 0.2 g), *n* (%)		Control (n = 7), *n* (%)
		BID (n = 25)	QD (n = 11)	
Characteristics				
Age (years)	49.5 (15.3)	45.8 (12.8)	57.2 (16.6)	49.5 (18.3)
0–14	0	0	0	0
15–44	18 (42)	12 (48)	2 (18)	4 (57)
45–64	18 (42)	12 (48)	5 (46)	1 (14)
≥65	7 (16)	1 (4)	4 (36)	2 (29)
Female sex	22 (51)	14 (56)	5 (46)	3 (43)
Underlying medical condition characteristics				
Overweight (BMI = 25–29.9)	6 (13.9)	5 (20)	1 (9)	0
Hypertension	4 (9.3)	2 (8)	1 (9)	1 (14.2)
Diabetes	4 (9.3)	2 (8)	1 (9)	1 (14.2)
Coronary heart disease	3 (6.9)	0	2 (18.1)	1 (14.2)
HBV carrier	2 (4.6)	2 (8)	0	0
Chronic pyelonephritis	1 (2.3)	1 (4)	0	0
Atherosclerosis	1 (2.3)	1 (4)	0	0
Gout	1 (2.3)	1 (4)	0	0
Cerebral Infarction	1 (2.3)	0	1 (9)	0
Coronary Sclerosis	1 (2.3)	0	1 (9)	0
Hyperlipidemia	1 (2.3)	0	1 (9)	0
Urethral carcinoma	1 (2.3)	0	1 (9)	0
pancreatic head carcinoma	1 (2.3)	0	1 (9)	0
Emphysema	1 (2.3)	0	0	1 (14.2)
Stage classification				
Critical	1 (2.3)	0	1 (9)	0
Severe	7 (16.3)	6 (24)	1 (9)	0
Ordinary	35 (81.4)	19 (76)	9 (82)	7 (100)
Midterm outcomes				
Remission	38 (88)	25 (100)	9 (82)	4 (57)
Exacerbation	5 (12)	0	2 (18)	3 (43)
Endpoint (March 20, 2020)				
Cured and discharged rate	43 (100)	25 (100)	11 (100)	7 (100)
Death rate	0	0	0	0

The special case E37 who used Ibuprofen was also discharged on February 19, 2020.

a Data are mean (SD) or n (%); reduced denominators indicate missing data. Percentages may not total 100 because of rounding. BID, twice a day; QD, once a day.

b There was a special case (named E37 in **Supplementary Table S1**) that first took Ibuprofen and continued for 17 days without switching to Celebrex. Compared to the Celebrex treatment, this patient experienced that ibuprofen also aided in the recovery of coronavirus disease 2019 infection, but had an overall less curative effect than Celebrex. Therefore, we did not put this special case into **Table 1**.

### Clinical Information and Celebrex Usage

Clinical information including the physical, laboratory tests and chest CT of COVID-19 patients was collected.

The patients in experimental group were treated with Celebrex (celecoxib, Pfizer, Dalian, China) in combination with routine treatments suggested by the guideline. The usage and dose of Celebrex was once or twice a day (0.2 g/time) for 7–14 days by oral. The dosage or duration of medication was subject to change based on each individual case. Routine treatments were according to the national guideline, including isolation, nursing, bed rest, symptomatic and supportive treatment, antibiotics, antiviral medication, glucocorticoids, oxygen therapy, and/or assisted breathing.

Based on the comparison of sequential chest CT images, as well as the changes of symptoms and laboratory test results, the clinical outcomes will be classified into three categories: remission, constant, and exacerbation. Remission was defined as dissipating/clarifying of the mass opacities in chest CT images; decreasing the D-dimer, C-reactive protein (CRP), serum alanine aminotransferase, and aspartate aminotransferase levels; and improvement of lymphopenia and neutrophilia; the constant and exacerbation outcomes were evaluated accordingly with the changes of those parameters. The length of Celebrex treatment was determined by the individual conditions. The discharge standards according to the national guideline are listed as following: 1) with normal body temperature for more than 3 days, 2) with significantly recovered respiratory symptoms, 3) lung imaging shows obvious absorption and recovery of acute exudative lesion, 4) with negative results of the nucleic acid tests of respiratory pathogens for consecutive two times (sampling interval at least 1 day).

### The Measurement of Urine Prostaglandin E_2_ by Mass Spectrometry

Agilent 1290 Infinity II high-performance liquid chromatograph was used in conjunction with Agilent 6470 triple quadrupole mass spectrometer for PGE_2_ measurement with modifications to the previous method (Cao et al., 2008). The sample was mixed with 3x volume of ethanol to inactivate the virus, and the resulted supernatant was mixed with an equal volume of methanol. After being centrifuged at 13,000 g for 15 min, the supernatant was mixed with 2× volumes of ddH_2_O (0.1% HCOOH) and analyzed by Agilent LC-QQQ 6470 with a 1290 high-performance liquid chromatograph. PGE_2_ was separated by using Zorbax Eclipse Plus C18 3.0 × 150 mm (1.8 *μ*m) with a 10-min linear gradient acetonitrile (0.1% HCOOH), and measured by monitoring *m/z* 351.1 to 271.3 under MRM with negative ion.

### Statistical Analysis

The differences between two groups of data were compared by Student’s t test, and the statistical results were expressed by mean ± standard error (mean ± SEM). Statistical analysis was conducted by using GraphPad Prism 7 software. *p* < 0.05 was considered statistically significant.

## Results

**FIGURE 1:**
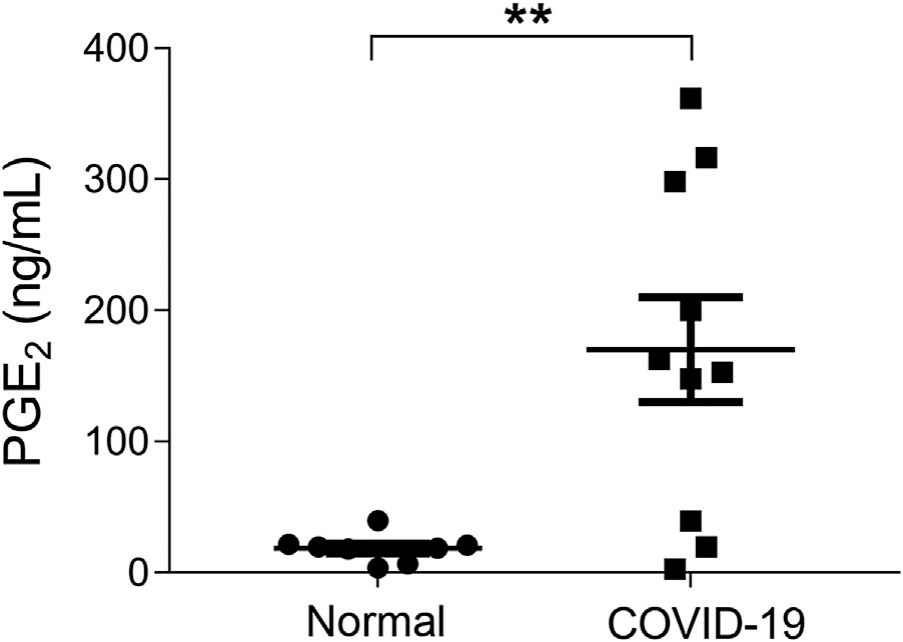
Urinary prostaglandin E_2_ (PGE_2_) levels of coronavirus disease 2019 (COVID-19) patients were significantly increased. Urinary prostaglandin E_2_ concentrations of COVID-19 patients, who were hospitalized within 2 days, were significantly higher than the health individuals (170 ± 40 ng/ml vs. 18.8 ± 3.8 ng/ml, *p* < 0.01). **p < 0.01.

The concentrations of PGE_2_ in urine samples were determined by a method of mass spectrometry (Supplementary Figure S1). Our data showed that the PGE_2_ levels in COVID-19 patients, who were hospitalized within 2 days, were significantly higher than the ones of healthy individuals (170 ± 40 ng/ml vs 18.8 ± 3.8 ng/ml, *p* < 0.01) (Figure 1). We determined that the normal threshold of PGE_2_ concentration in urine is lower than 20 ng/ml and that 100 ng/ml is considered to be significant as a risk line.

Since the PGE_2_ was mainly generated by COX-2, a COX-2–specific inhibitor (Celebrex) was used to treat COVID-19 patients based on the routine treatment. On March 19, 2020, 25 cases (six severe and 19 ordinary) were given full dose (0.2 g, twice a day) of Celebrex, and all cases showed improved outcomes after discontinuation. There were 11 cases (two severe/critical and nine ordinary) that had received half dose (0.2 g, once a day) of Celebrex, and all the ordinary cases showed improvement, while the 2 severe/critical cases were exacerbated after discontinuation of treatment. Whereas in control group (*n* = 7), four cases were improved and three cases were exacerbated at day 10 after admission in hospital (Table 1; Supplementary Table S1). There was a special case in the experimental group (case E37 in Supplementary Table S1), who was given ibuprofen (COX-1 and COX-2 inhibitor) in the first day, and then continued for 17 days without switching to Celebrex (COX-2 selective inhibitor). Compared to the Celebrex treatment, ibuprofen also promoted the recovery of COVID-19; however, the overall curative effect was less than Celebrex. Considering the value of this information, this special case was included and specifically isolated in Supplementary Table S1 (the red highlight). Our results indicated that Celebrex treatment with a conventional dose (0.2 g, twice a day) might effectively promote the recovery of ordinary and severe cases of COVID-19.

**FIGURE 2:**
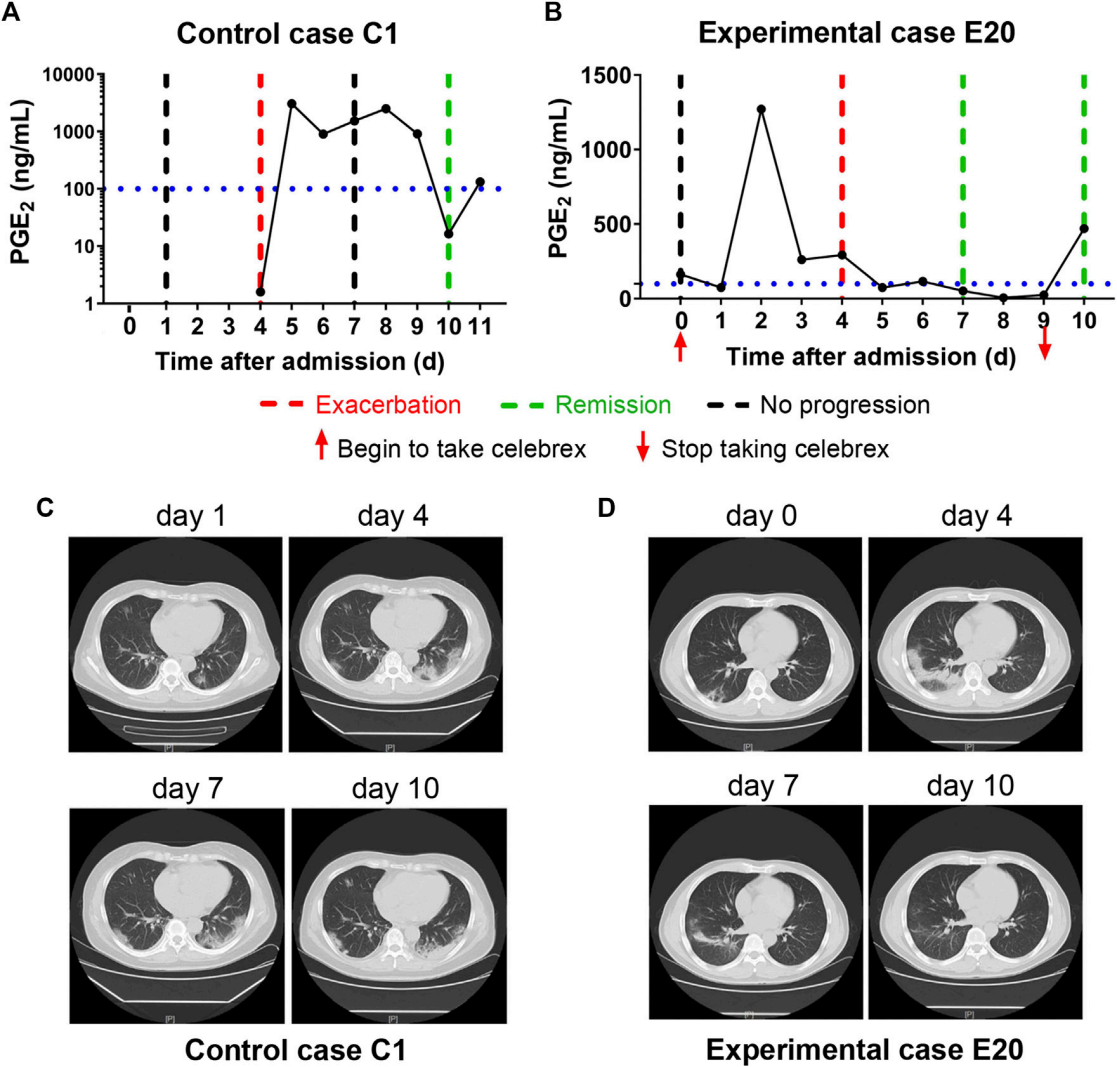
Celebrex treatment accelerated the recovery of those ordinary coronavirus disease 2019 (COVID-19) patients and prevented the progression toward severe stage. **(A)** Control case C1, the dynamic changes of the urinary prostaglandin E_2_ were correlated with the COVID-19 prognosis; **(B)** experimental case E20, the dynamic reduction of prostaglandin E_2_ was matched with the improvements of COVID-19 conditions; **(C,D)** representation of sequential chest CT images illustrated therapeutic outcomes of these two cases, respectively.

It has been reported that 15.7% of the ordinary COVID-19 cases progressed to a severe stage under routine treatments (Guan et al., 2020). With Celebrex treatment, none of the 29 ordinary cases progressed to a severe classification (Supplementary Table S1). Two ordinary cases were chosen to represent the control (case C1) and the experimental group (case E20), respectively (Figure 2). The PGE_2_ level of C1 remained at a high level (>1,000 ng/ml) during days 5–9 (Figure 2A), while the case E20 treated with Celebrex decreased steadily from over 1,000 ng/ml (day 2) to 100 ng/ml (day 3–4) (Figure 2B). Except the improvements of symptoms and laboratory tests, the chest CT images (at day 4–10) showed that the improvement of pulmonary opacification and pneumonia of case E20 was faster than that of the control group C1 case in the same period (Figures 2C,D). It suggested that Celebrex might promote the recovery process of ordinary COVID-19 patients.

**FIGURE 3:**
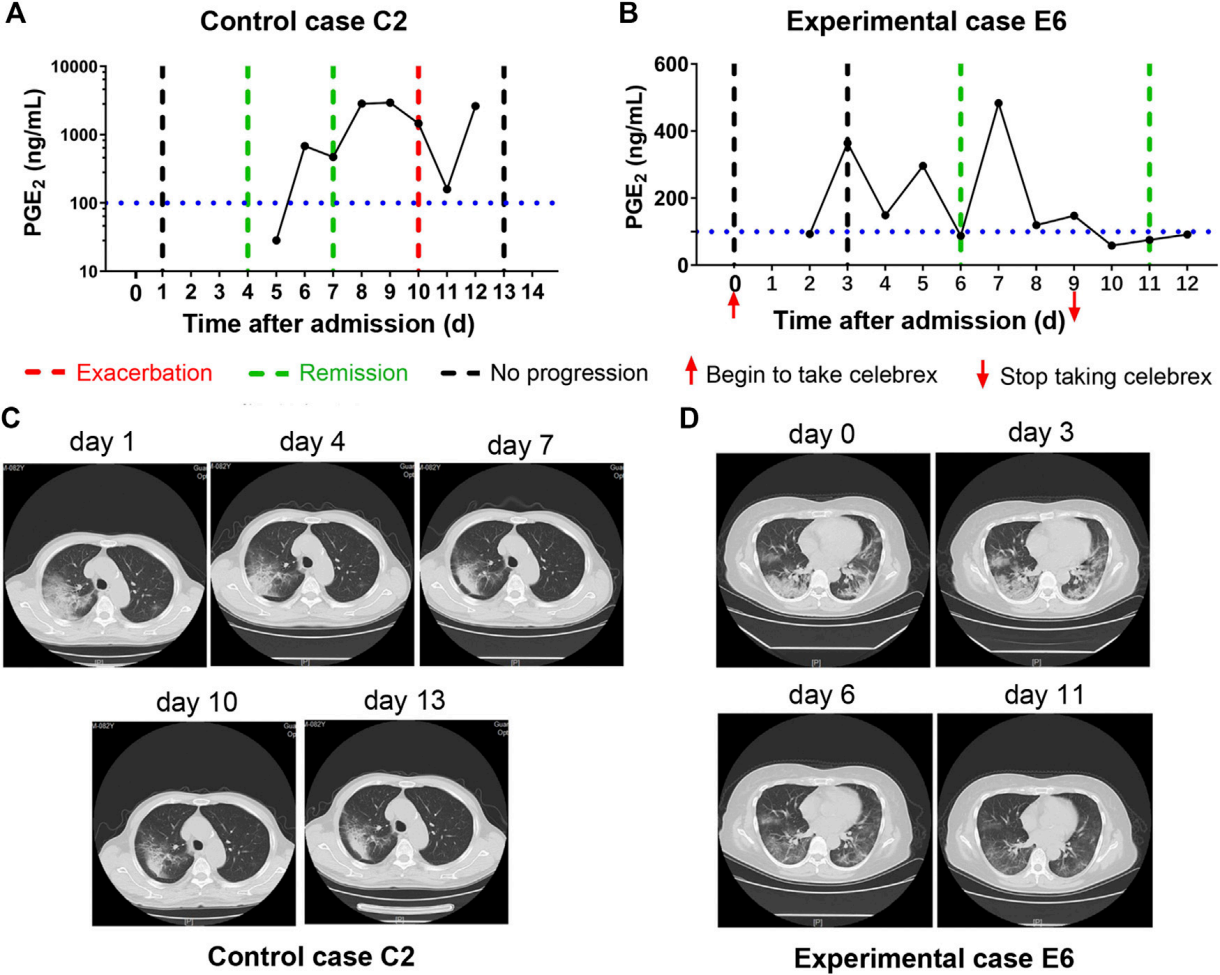
Celebrex treatment promoted the improvement of severe stage of coronavirus disease 2019 (COVID-19) and blocked the progression toward critical stage. **(A)** Control case C2, the dynamic changes of the urinary prostaglandin E_2_ were correlated with its COVID-19 prognosis; **(B)** experimental case E6, the dynamic reduction of prostaglandin E_2_ was matched with the improvements of COVID-19 conditions; **(C,D)** representation of sequential chest CT images illustrated therapeutic outcomes of these two cases, respectively.

In addition, none of the severe COVID-19 cases with full dose of Celebrex treatment progressed to critical illness. The comparison of the control (case C2) and experimental cases (case E6) with similar severe pulmonary opacification diagnosed by CT imaging was illustrated (Figure 3). The PGE_2_ levels of case C2 fluctuated around 1,000 ng/ml at days 5–12 after admitted to hospital with routine treatment (Figure 3A). In contrast, the PGE_2_ levels of case E6 in the Celebrex group were steady decrease, and remained at lower level than 100 ng/ml (Figure 3B). Indeed, the chest CT images (at days 3, 6, and 11) of case E6 showed that the ground glass–like opacities were clarified continually, and significantly faster than the control case C2 (Figures 3C and D). It suggests that Celebrex might reverse the progress of severe COVID-19 and prevented the progression to a critical stage.

**FIGURE 4:**
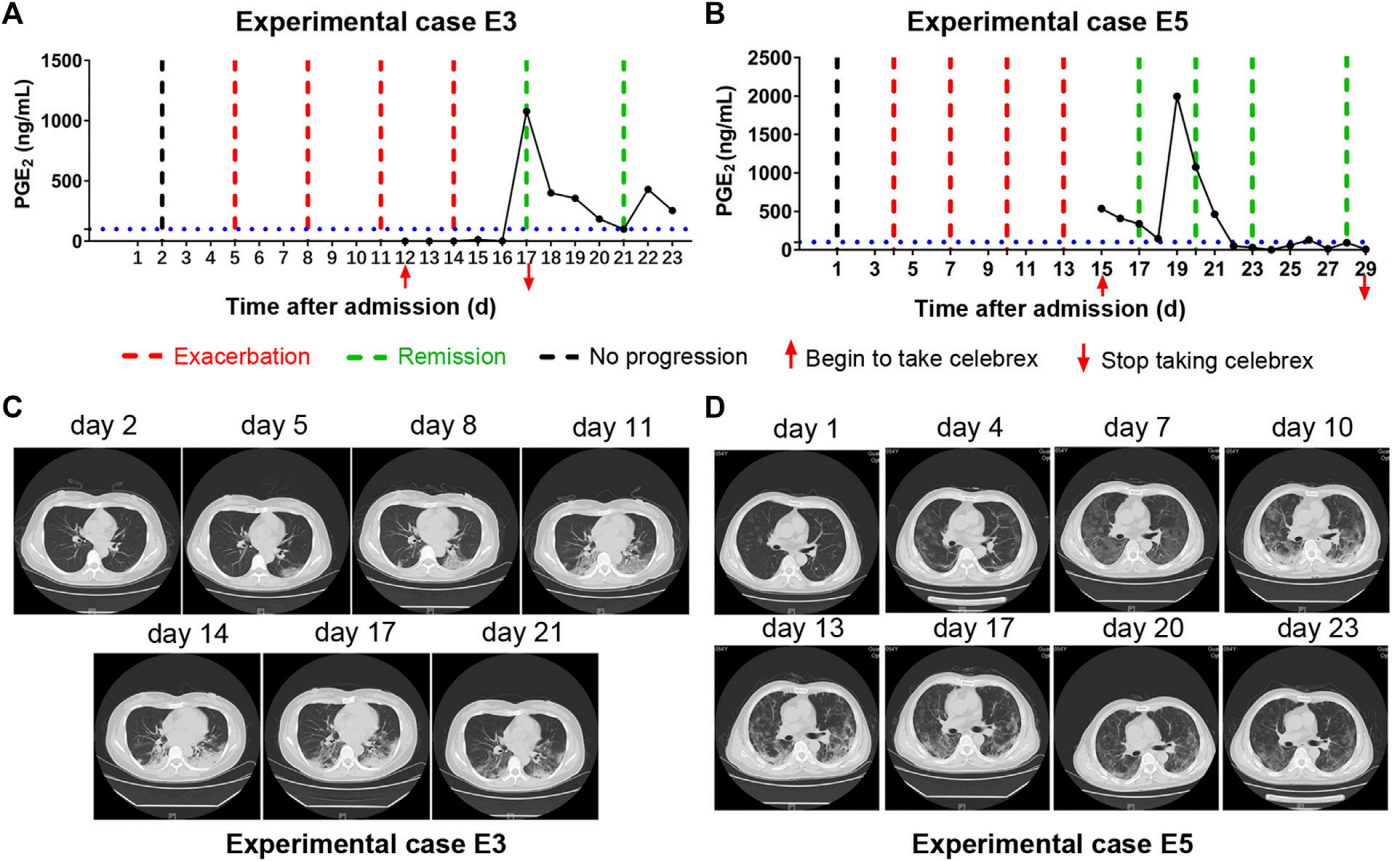
Celebrex intervention reversed the progressed severe stage under routine treatment. **(A,B)** Two ordinary cases received routine treatment for 12 and 15 days, respectively, and the conditions gradually progressed into severe stage; upon the Celebrex treatment, the prostaglandin E_2_ levels were decreased along with the improvements of coronavirus disease 2019; **(C,D)** representation of sequential chest CT images illustrated therapeutic outcomes of these two cases under Celebrex intervention, respectively.

Moreover, there were two patients (experimental cases E3 and E5), who were hospitalized and received routine treatment for 12 and 15 days, respectively, progressed from ordinary to severe illness. After taking Celebrex based on routine treatment, their PGE_2_ levels were controlled and the pneumonia were gradually improved (Figure 4). These findings indicated that Celebrex may also be effective on patients who progressed from ordinary into severe type under routine therapy.

Altogether, our findings suggested that the increase of PGE_2_ may be critical for the progression of COVID-19, and Celebrex intervention may effectively promote the recovery of ordinary and severe types of COVID-19.

## Discussion

ARDS is the leading cause of death of the critical SARS-CoV, MERS-CoV, and SARS-CoV-2–infected patients. Autopsy reports indicate that the pathological manifestation of lung injury were diffuse alveolar injuries, including fibrin mucus exudation, ground-glass edema membrane formation, and alveolar epithelial cell detachment (Ding et al., 2003; Ng et al., 2016; Zhu et al., 2019). However, the pulmonary fibrosis and consolidation caused by SARS-CoV-2 was less severe than SARS-CoV, but the alveolar mucous secretion was more severe (Liu et al., 2020). Therefore, it was speculated that the COVID-19 patient's alveolar blood and gas exchange were seriously blocked, which led to ARDS. The patient eventually died from respiratory and multi-organ failures.

Prostaglandin is a class of small lipid molecules transformed from arachidonic acid by COX-1 and COX-2, and PGE_2_ is one of the most active molecules (Park and Christman, 2006). The COX-2/PGE_2_ pathway plays a crucial role in mucus secretion, desensitization of the *β*-2 adrenergic receptor, and the matrix metalloproteinase-mediated airway remodeling, cough, fever, asthma, and other respiratory diseases (Rumzhum and Ammit, 2016). It has been reported that both N and S proteins of SARS-CoV could induce the expression of COX-2 in epithelial cells (Yan et al., 2006; Liu et al., 2007). The similarity of N and S protein sequences of SARS-CoV-2 and SARS-CoV was 90.6 and 75.8%, respectively. It was found that both viruses infected host cells by using the S protein to bind angiotensin I converting enzyme 2 receptor (Hoffmann et al., 2020). Thus, it suggests that SARS-CoV-2 might also have the ability to induce the expression of COX-2 in lung epithelial cells.

We confirmed that the urine PGE_2_ concentration of COVID-19 patients was significantly higher than that of healthy individuals. Celebrex, a specific inhibitor of COX-2, effectively decreased the level of urinary PGE_2_ and promoted the recovery of COVID-19. However, we also found that, in some cases, discontinuation of Celebrex might lead to increased PGE_2_ and pneumonia relapse, such as experimental cases E1 and E9, whose PGE_2_ levels were rebounded and increased quickly accompanied with slightly worsened pulmonary opacification (Supplementary Figure S2). These observations suggested that the duration of Celebrex treatment should be determined according to each patient's condition to reduce the risk of disease relapse, deterioration, and pulmonary fibrosis.

The half-life of PGE_2_ *in vivo* is about 1 min (Hamberg and Samuelsson, 1971). After going through the lung, liver, and kidney organs *via* blood circulation, up to 90% of PGE_2_ will be degraded. Extremely high concentrations of PGE_2_ (up to 2,500 ng/ml) were detected in the urine of COVID-19 patients, such as control cases C1 and C2 (Figures 2, 3), so it is conceivable that the PGE_2_ levels in lung tissues and blood could be much higher. This kind of “prostaglandin storm” occurs in the very early and progressive stages of COVID-19, while very few immune cytokines could be detected in blood or urine during this early period, which could occur at the critical stage.

Currently, Zheng et al reported that in an influenza virus–infected mouse model of H5N1, the survival rate of mice treated with zanamivir alone (antiviral drug) was 13.3%, while combining zanamivir with Celebrex (COX-2 inhibitor) or with mesalazine (COX inhibitor) could improve the survival to about 20%. However, a combination of these three drugs significantly reduced viral load and increased survival up to 53.3% (Zheng et al., 2008). Now there is no specific drug for the treatment of COVID-19 today, we supposed that Celebrex combined with antivirus and other anti-inflammatory drugs treatment might be a good strategy for COVID-19 treatment.

There are no reports on whether Celebrex is involved in inhibiting SARS-CoV-2 replication. However, some studies have found that COX-1 and COX-2 inhibitors have the potential to inhibit replication of other subtypes of coronavirus. For example, Raaben et al reported that indomethacin and curcumin (COX nonselective inhibitors) inhibited the synthesis of RNA, protein, and production of virus particles of mouse hepatitis coronavirus in a dose-dependent manner. *In vitro* experiments showed that both SC-560 (COX-1 inhibitor) and NS-398 (COX-2 inhibitor) reduced mouse hepatitis virus (coronavirus) infection by 65–75% at concentrations that were nontoxic to the cells (Raaben et al., 2007). Santoro *et al* reported that indomethacin directly inhibited the production of SARS-CoV and CCoV virus particles, reduced cell infection *in vitro* and *in vivo* (Amici et al., 2006).

Since this study was started at the beginning of the outbreak of COVID-19 which was an emergency situation, the first 15 enrolled COVID-19 patients were randomized/controlled assigned into the experimental or control group. The midterm evaluations revealed that therapeutic efficacy of Celebrex was significant greater than that of controls. According to the requirements of medical ethics for clinical study, those with aggravating conditions in the control group were transferred into the experimental group, and the subsequently enrolled patients were recruited directly into the experimental group. In addition, we did not have much choice about the age, gender, or severity of the disease because there were too few COVID-19 cases in Guangdong Province at the beginning of the outbreak.

Moreover, most patients were ordinary type of COVID-19 during the period of this clinical study. Supplementary Table S1 summarized that the COVID-19 stage distribution of the enrolled total 44 cases were 36 ordinary, 7 serve, and 1 critical type at the recruitment time point. Furthermore, few more critical patients did treat with Celebrex and had good outcome, but without continuing daily PGE2 measurements caused by the emergency situation; therefore, those data were not included in the final analysis.

In Celebrex-treated group, there were three patients (two female and one male) noticed with “side effects” including sweating, mental disorder, and abnormal liver function, respectively. And those side effects are very rarely reported during over three-decade clinical application of Celebrex. The details of these three cases are as following:

Case E3 (age 47, female, severe type): she had fever, and the Celebrex administration brought down the fever accompanying with sweating. The comprehensive judgment of the doctor team is that it is not a real “side effect,” but a response of reducing body temperature. We did discuss with this patient and suggested her to drink more water with a little salt and continue the Celebrex treatment. The patient hesitated with worries; therefore, Celebrex was withdrawn, while the PGE2 sequentially increased from the normal region to over 350 ng/ml in the following days.

Case E5 (age 54, male, severe type): Celebrex administration period was from February 13 to 27. During February 23 to 26, mental disorders were observed of this patient, and reviewing analysis found that this particular patient also had administration of chloroquine phosphate (0.5 g per day) from February 13 to 22.

Case E19 (age 41, female, ordinary type): this patient enrolled into the Celebrex treatment group from February 11 to 25; just on the final day of Celebrex, a minor increase of ALT and AST was noticed and recovered to normal in the following week. The symptoms of this case were more serious among those ordinary type patients; the opinion from the doctors was that SARS-CoV-2 infection might be the most likely reason leading to liver damage in this case. But we could not rule out the possibility from other drugs used in this period. Overall, all three patients were cured of COVID-19 and remission from hospital without any sequela.

In addition, a great number of evidence globally indicate that the obesity, diabetes, and cardiovascular conditions worsen the recovery process of COVID-19. Therefore, body weight, height, and basal medical conditions of all those 44 cases were included, and body mass index (BMI) was calculated (Supplementary Table S1). We found that there were no obesity condition either in the Celebrex or control group; there was no overweight case in the control group, but six cases (BMI: 25–30) occupied 16% (6/37) of the Celebrex group. Moreover, there were 13 cases (35%, 13/37) in the Celebrex group that had other medical conditions. Those diseases are included as serious as pancreatic carcinoma, urethral carcinoma, atherosclerosis, cerebral infarction, diabetes, and chronic pyelonephritis, as mild as hyperlipidemia, hypertension, gout, and HBV carrier. In the control group, there was 1 case with emphysema, hypertension, and coronary heart disease.

Elder plus medical conditions: in terms of the age, there were four patients aged over 70 years (10%, 4/37), in which three (75%, 3/4) of them had serious conditions such as cancer, coronary heart disease, or diabetes; one case was severe type COVID-19; 10 cases aged over 60 years (27%, 10/37) in which six cases (60%, 6/10) were with other diseases.

It is noteworthy that more complications, severity and fatality rate were observed in the COVID-19 group of elders and with comorbidities; however, this phenomenon did not appear in this particular Celebrex adjunctive treatment study. This phenomenon was also observed in another Celebrex adjunctive therapy study for COVID-19 reported from Beloit Memorial Hospital, United States (Tomera et al., 2020). There were 25 hospitalized patients enrolled this study, in which 36% of them were with life-threatened high lactate dehydrogenase, 80% with obesity, 44% cardiovascular disease, 40% diabetes, and 32% renal disease and other medical conditions. The outcome of Celebrex treatment showed 100% survival, and quick and substantial clinical improvements of recovery.

Taken together, COX-2 overexpression accompanied with PGE_2_ accumulation may be a key in the molecular pathology of COVID-19. Celebrex, a specific COX-2 inhibitor, may be an effective drug for the treatment on COVID-19. However, our study was not a rigorous randomized, double-blind, and controlled clinical trial. Another well-designed large-scale clinical trial is needed to validate this hypothesis. Our study may provide useful information for the treatment of COVID-19.

## Data Availability Statement

The datasets used and/or analyzed during this study are available from the corresponding authors on reasonable request.

## Ethics Statement

The studies involving human participants were reviewed and approved by Medical Ethics Committee of Guangzhou Eighth People's Hospital (Approve number: AF/sc-02/01.6). The patients/participants provided their written informed consent to participate in this study.

## Author Contributions

LYX, FZ, and JW conceived and designed the study. YC, KY, ST, and FW contributed to the literature search. WH, YC, KY, ST, FW, JT, XuC, JZ, YX, YF, ZY, TC, PP, and QT contributed to data collection. WH, YC, KY, ST, FW, and JT contributed to data analysis. WH, XuC, XiC, and PP contributed to data interpretation. WH, YC, KY, ST, and JT contributed to the figures. YC, KY, ST, FW, JT, and LYX contributed to writing of the report.

## Funding

This study was supported by the Emergency and Special Research Project for Prevention and Control of COVID-19 from Guangdong Province (2020B111117001), the National Key Research and Development Program of China (2020YFC0842400), Guangzhou Regenerative Medicine and Health Guangdong Laboratory (2020GZR110106005, 2018GZR110105011), National Major Scientific and Technological Special Project (2018ZX10102–001), National Natural Science Foundation of China (31871379), Science and Technology Project of Guangdong Province (2018A050506070), Guangzhou Science and Technology Project (201704020212), Chinese Postdoctoral Science Foundation (2019M663142, 2019M652848).

## Conflict of Interest

The authors declare that the research was conducted in the absence of any commercial or financial relationships that could be construed as a potential conflict of interest.
