# Supplementary material for: Celebrex Adjuvant Therapy on Coronavirus Disease 2019: An Experimental Study
Source: Front Pharmacol. 2020 Nov 6;11:561674. doi: 10.3389/fphar.2020.561674 (PMC7703865; doi:10.3389/fphar.2020.561674)
Supplement: Supplementary file 1 [file DataSheet1_v1.DOCX]

*Supplementary Material*

# Supplementary Table

| **Supplementary Table 1. Detail information of Celebrex adjunctive therapy for COVID-19 patients** | | | | | | | | | | | | | | | |
| --- | --- | --- | --- | --- | --- | --- | --- | --- | --- | --- | --- | --- | --- | --- | --- |
| **Case No.** | **Gender** | **Age** | **Body weight (kg)** | **Height**  **(cm)** | **BMI ¤** | **Other conditions ∏** | **Hospitalized date** | **Stage Classification †** | **Celebrex adjuvant therapy** | | | | | | **Discharged date** |
|  |  |  |  |  |  |  |  |  | Enrolled date | Administration  by oral # | | Ended date | Outcomes after discontinuation of Celebrex | Side effects |  |
| **Celebrex group (Full dose)** | | | | | | | | | | | | | | | |
| E1 | Female | 71 | 52 | 152 | 22.5 | Non | 2020/2/2 | **Severe** | 2020/2/4 | 0.2g *bid* | | 2020/2/15 | Remission | Non | 2020/3/19 |
| E2 | Female | 60 | 55 | 143 | 26.9 | CP | 2020/2/2 | **Severe** | 2020/2/4 | 0.2g *bid* | | 2020/2/15 | Remission | Non | 2020/2/17 |
| E3 | Female | 47 | 57 | 154 | 24.0 | Non | 2020/1/23 | **Severe** | 2020/2/4 | 0.2g *bid* | | 2020/2/9 | Remission | Sweating | 2020/2/15 |
| E4 | Male | 64 | 72 | 172 | 24.3 | Non | 2020/1/31 | **Severe** | 2020/2/4 | 0.2g *bid* | | 2020/2/15 | Remission | Non | 2020/3/4 |
| E5 | Male | 54 | 62 | 168 | 22.0 | HBV carrier | 2020/1/29 | **Severe** | 2020/2/13 | 0.2g *bid* | | 2020/2/27 | Remission | Mental disorder ‡ | 2020/3/8 |
| E6 | Female | 58 | 48 | 145 | 22.8 | H, D | 2020/2/6 | **Severe** | 2020/2/6 | 0.2g *bid* | | 2020/2/15 | Remission | Non | 2020/2/18 |
| E7 | Male | 50 | 66 | 167 | 23.7 | Gout | 2020/1/23 | Ordinary | 2020/2/13 | 0.2g *bid* | | 2020/2/21 | Remission | Non | 2020/2/21 |
| E8 | Male | 26 | 68 | 173 | 23.7 | Non | 2020/1/28 | Ordinary | 2020/2/4 | 0.2g *bid* | | 2020/2/10 | Remission | Non | 2020/2/10 |
| E9 | Female | 63 | 52 | 151 | 22.8 | Non | 2020/1/29 | Ordinary | 2020/2/4 | 0.2g *bid* | | 2020/2/15 | Remission | Non | 2020/2/29 |
| E10 | Male | 33 | 68 | 171 | 23.3 | HBV carrier | 2020/2/15 | Ordinary | 2020/2/15 | 0.2g *bid* | | 2020/2/25 | Remission | Non | 2020/2/25 |
| E11 | Female | 43 | 66 | 170 | 22.8 | Non | 2020/1/30 | Ordinary | 2020/2/4 | 0.2g *bid* | | 2020/2/17 | Remission | Non | **2020/2/18 ¶** |
| E12 | Male | 33 | 71 | 174 | 23.5 | Non | 2020/1/31 | Ordinary | 2020/2/9 | 0.2g *bid* | | 2020/2/25 | Remission | Non | 2020/2/28 |
| E13 | Female | 62 | 58 | 151 | 25.4 | H | 2020/2/5 | Ordinary | 2020/2/10 | 0.2g *bid* | | 2020/2/13 | Remission | Non | **2020/2/13 ¶** |
| E14 | Female | 37 | 55 | 154 | 23.2 | Non | 2020/2/7 | Ordinary | 2020/2/10 | 0.2g *bid* | | 2020/2/17 | Remission | Non | 2020/2/17 |
| E15 | Male | 43 | 67 | 172 | 22.6 | Non | 2020/2/8 | Ordinary | 2020/2/10 | 0.2g *bid* | | 2020/2/27 | Remission | Non | 2020/3/5 |
| E16 | Male | 44 | 62 | 165 | 22.8 | Non | 2020/2/8 | Ordinary | 2020/2/8 | 0.2g *bid* | | 2020/2/17 | Remission | Non | 2020/2/17 |
| E17 | Female | 31 | 54 | 158 | 21.6 | Non | 2020/2/8 | Ordinary | 2020/2/11 | 0.2g *bid* | | 2020/2/14 | Remission | Non | 2020/2/21 |
| E18 | Female | 33 | 50 | 149 | 22.5 | Non | 2020/2/9 | Ordinary | 2020/2/10 | 0.2g *bid* | | 2020/2/16 | Remission | Non | 2020/3/8 |
| E19 | Female | 41 | 49 | 155 | 20.4 | Non | 2020/2/10 | Ordinary | 2020/2/11 | 0.2g *bid* | | 2020/2/25 | Remission | liver dysfunction | 2020/3/7 |
| E20 | Male | 45 | 62 | 166 | 22.5 | Non | 2020/2/13 | Ordinary | 2020/2/13 | 0.2g *bid* | | 2020/2/27 | Remission | Non | 2020/3/3 |
| E21 | Male | 24 | 71 | 173 | 23.7 | Non | 2020/2/9 | Ordinary | 2020/2/11 | 0.2g *bid* | | 2020/2/18 | Remission | Non | 2020/3/5 |
| E22 | Female | 58 | 63 | 161 | 24.3 | A | 2020/2/9 | Ordinary | 2020/2/10 | 0.2g *bid* | | 2020/2/18 | Remission | Non | 2020/2/21 |
| E23 | Female | 45 | 61 | 154 | 25.7 | Non | 2020/2/15 | Ordinary | 2020/2/16 | 0.2g *bid* | | 2020/2/27 | Remission | Non | 2020/3/3 |
| E24 | Male | 47 | 71 | 168 | 25.2 | D | 2020/2/14 | Ordinary | 2020/2/15 | 0.2g *bid* | | 2020/2/27 | Remission | Non | 2020/3/3 |
| E25 | Female | 33 | 58 | 145 | 27.6 | Non | 2020/2/14 | Ordinary | 2020/2/15 | 0.2g *bid* | | 2020/2/24 | Remission | Non | 2020/2/26 |
| **Celebrex group (Half dose)** | | | | | | | | | | | | | | | |
| **E26 §** | Female | 55 | 46 | 151 | 20.2 | Non | 2020/1/26 | **Critical in ICU** | 2020/2/9 | 0.2g *qd* | 2020/2/16 | | **Exacerbation** | Non | 2020/3/14 |
| E27 | Male | 67 | 59 | 165 | 21.7 | H, CI, CS | 2020/1/30 | Ordinary | 2020/2/5 | 0.2g *qd* | 2020/3/2 | | Remission | Non | 2020/3/2 |
| E28 | Male | 73 | 63 | 168 | 22.3 | CHD | 2020/1/30 | Ordinary | 2020/2/6 | 0.2g *qd* | 2020/2/7 | | Remission | Non | 2020/2/25 |
| E29 | Female | 78 | 42 | 143 | 20.5 | Hyp | 2020/2/1 | Ordinary | 2020/2/4 | 0.2g *qd* | 2020/2/10 | | Remission | Non | 2020/2/27 |
| E30 | Male | 37 | 72 | 172 | 24.3 | Non | 2020/2/1 | Ordinary | 2020/2/5 | 0.2g *qd* | 2020/2/29 | | Remission | Non | 2020/2/29 |
| **E31 §** | Male | 57 | 63 | 167 | 22.6 | UC | 2020/2/1 | **Severe** | 2020/2/11 | 0.2g *qd* | 2020/2/14 | | **Exacerbation** | Non | 2020/2/29 |
| E32 | Female | 62 | 51 | 151 | 22.4 | Non | 2020/2/2 | Ordinary | 2020/2/4 | 0.2g *qd* | 2020/2/8 | | Remission | Non | 2020/2/10 |
| E33 | Male | 30 | 70 | 170 | 24.2 | Non | 2020/2/3 | Ordinary | 2020/2/5 | 0.2g *qd* | 2020/2/14 | | Remission | Non | 2020/2/20 |
| E34 | Male | 59 | 67 | 163 | 25.2 | Non | 2020/2/3 | Ordinary | 2020/2/5 | 0.2g *qd* | 2020/2/10 | | Remission | Non | 2020/3/2 |
| E35 | Female | 53 | 44 | 143 | 21.5 | Non | 2020/2/3 | Ordinary | 2020/2/5 | 0.2g *qd* | 2020/2/14 | | Remission | Non | 2020/2/24 |
| E36 | Female | 81 | 41 | 140 | 20.9 | D, CHD, PHC | 2020/2/4 | Ordinary | 2020/2/10 | 0.2g *qd* | 2020/2/12 | | Remission | Non | 2020/2/21 |
| **E37 *** | Male | 35 | 68 | 172 | 23.0 | Non | 2020/1/29 | Ordinary | 2020/2/2 | Ibuprofen suspension 8ml *bid* | 2020/2/19 | | Remission | Non | **2020/2/19 ¶** |
| **Control group** | | | | | | | | | | | | | | | |
| C1 | Female | 65 | 53 | 146 | 24.9 | Non | 2020/2/2 | Ordinary | / | / | / | | **Exacerbation** | / | 2020/2/15 |
| C2 | Male | 82 | 63 | 161 | 24.3 | E, H, D, CHD | 2020/2/1 | Ordinary | / | / | / | | **Exacerbation** | / | 2020/2/28 |
| C3 | Female | 50 | 54 | 152 | 23.4 | Non | 2020/2/2 | Ordinary | / | / | / | | Remission | / | 2020/2/12 |
| C4 | Female | 33 | 58 | 161 | 22.4 | Non | 2020/2/2 | Ordinary | / | / | / | | **Exacerbation** | / | 2020/2/16 |
| C5 | Male | 43 | 74 | 175 | 24.2 | Non | 2020/2/1 | Ordinary | / | / | / | | Remission | / | 2020/2/14 |
| C6 | Male | 30 | 70 | 173 | 23.4 | Non | 2020/2/6 | Ordinary | / | / | / | | Remission | / | 2020/2/13 |
| C7 | Male | 44 | 68 | 171 | 23.3 | Non | 2020/2/1 | Ordinary | / | / | / | | Remission | / | 2020/2/8 |

**¤** BMI: Body Mass Index.

**∏ Non:** no basal diseases before SARS-CoV-2 infection; **CP:** Chronic Pyelonephritis; **HBV carrier:** Hepatitis B Virus carrier; **H:** Hypertension; **D:** Diabetes; **A:** Atherosclerosis; **CI:** Cerebral Infarction; **CS:** Coronary Sclerosis; **CHD:** Coronary heart disease; **Hyp:** Hyperlipidemia; **UC:** Urethral carcinoma; **PHC:** pancreatic head carcinoma; **E:** Emphysema.

**†** Severity of illness, **mild:** the clinical symptoms are mild and no pneumonia manifestation can be found in CT imaging; **Ordinary:** Fever and respiratory tract symptoms, etc. and pneumonia manifestation can be seen in CT imaging; **Severe:** Meeting any of the following: 1) Respiratory distress, RR≥30 breaths/min; 2) Oxygen saturation ≤ 93% at a rest state; 3) Arterial partial pressure of oxygen (PaO2)/oxygen concentration (FiO2) ≤ 300mmHg; **Critical:** Meeting any of the following: 1) Respiratory failure occurs and mechanical ventilation is required; 2) Shock occurs; 3) Complicated with other organ failure that requires Intensive care unit (ICU) care.

**#** *bid*: twice a day; *qd*: once a day.

**‡** Case E5 (age 54, Male, severe type), the Celebrex administration period was from February 13 to 27. During February 23 to 26, mental disorders were observed of this patient, and reviewing analysis found that this particular patient also had administration of chloroquine phosphate (0.5 g per day) from February 13 to 22.

¶ The case E11, E13 and E37 in the experimental group had the SARS-CoV-2 returned to be positive after discharged and re-hospitalized on February 22, 21 and 28 respectively. Recently, three of them were discharged on March 4, 5 and 6.

**§** The case E26 and E31 in the experimental group were already in the ICU while received the half dose and 8 or 4 days Celebrex treatment, respectively.

***** Celebrex group case E37 was used ibuprofen treatment as a special case, and experienced less therapeutic effect comparison to Celebrex.

## Supplementary Figures

**
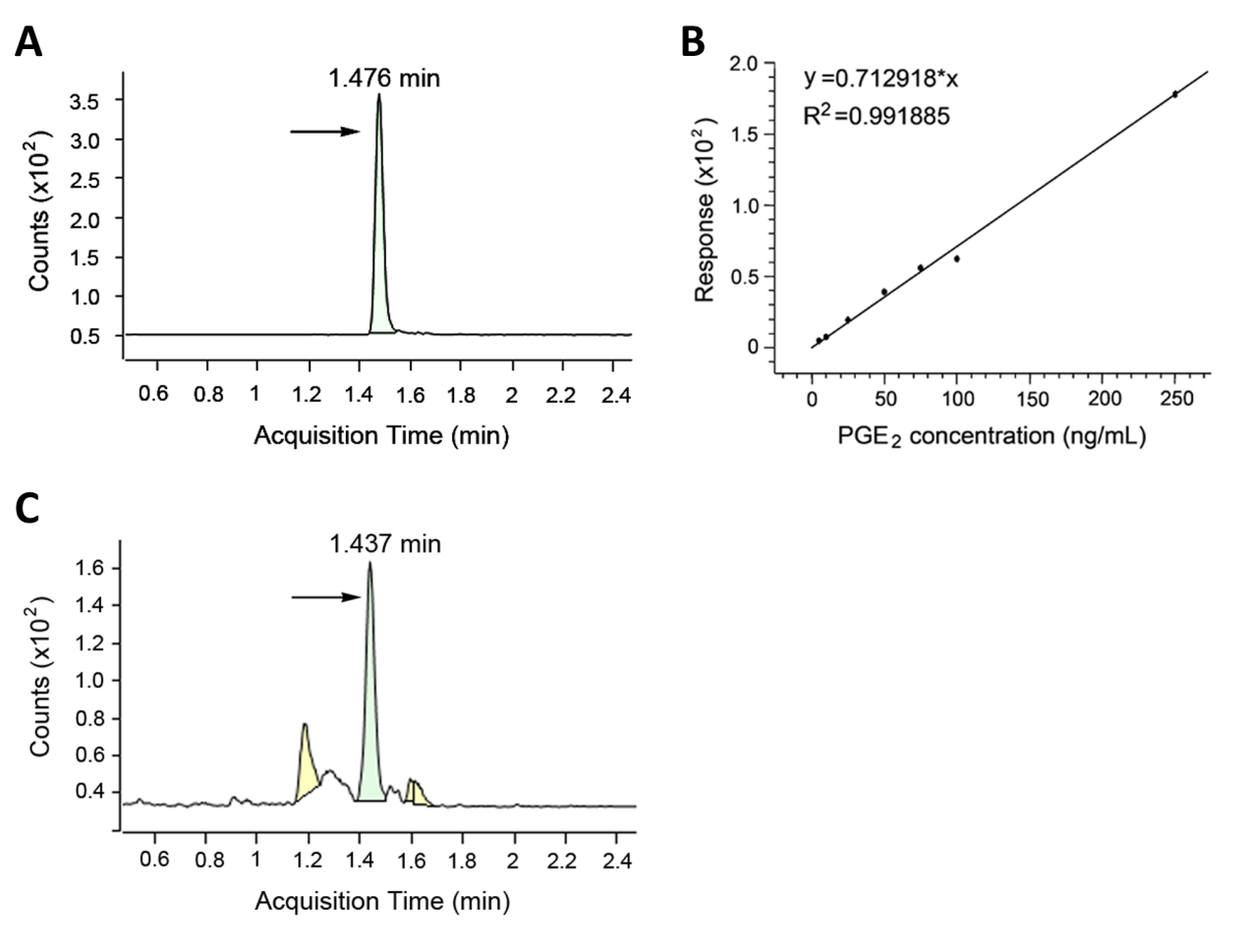
**

**Supplementary Figure 1: The measurement of urinary PGE_2_ in COVID-19 patients by mass spectrometry.** A: The specific peak of PGE_2_ detected by liquid mass spectrometry (the acquisition time was 1.476 min); B: Standard curve of PGE_2_ was linearized (the goodness of fit was R^2^ > 0.99); C: The urine samples of COVID-19 patients showed a specific peak of PGE_2_ (the acquisition time was 1.437 min), and the integral area of this specific peak represented the concentration of PGE_2_ (arrow).


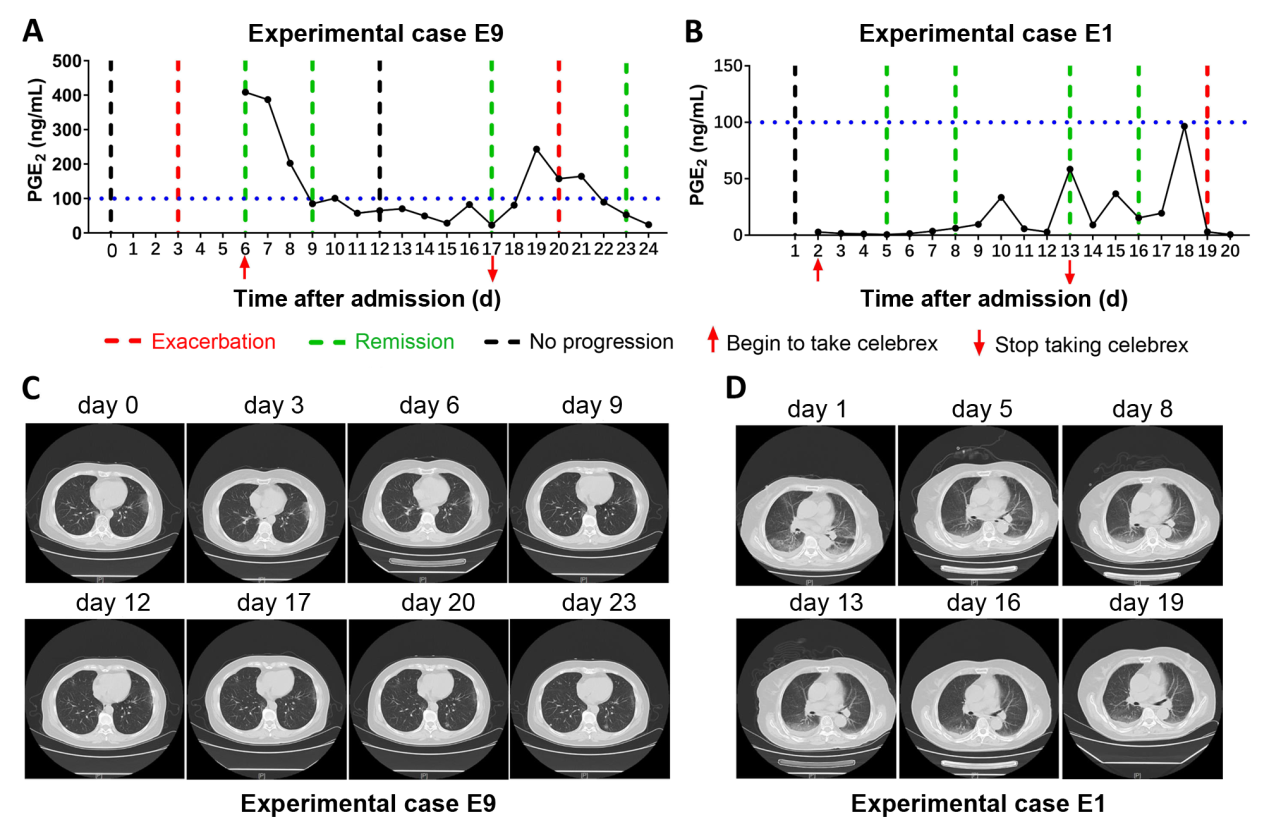


**Supplementary Figure 2. Withdraw of Celebrex caused PGE_2_ elevation and COVID-19 condition fluctuation.** A and B: Two represented cases upon the Celebrex termination (case E9 on day 17; case E1 on day 13), followed with increased urinary PGE_2_ (case E9 from day 17-20; case E1 from day 13-18); C and D: The representation of the chest CT images (case E 9 on day 19; case E1 on day 20) illustrated exacerbated outcomes upon withdraw of Celebrex.


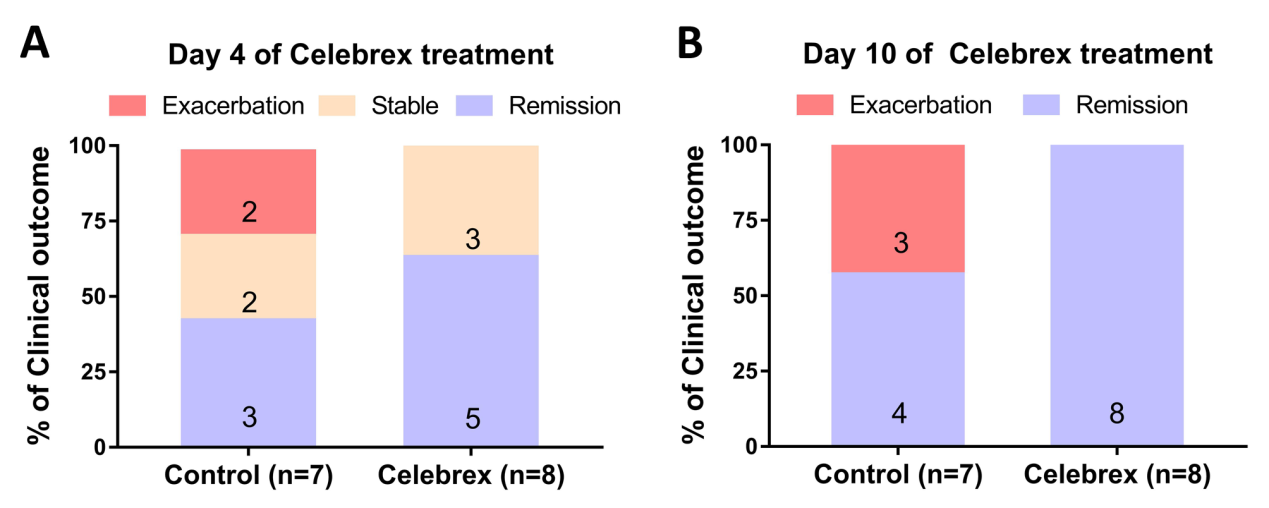


**Supplementary** **Figure 3. Mild-term outcome assessments of the first recruited 15 cases.** Based on the CT diagnoses results, as well as the changes of symptoms and laboratory test results, the overall mild-term of therapeutic efficacy of Celebrex group was significantly better than that of control group. Assessments were performed on day 4 (A) and day 10 (B) of Celebrex treatment.
